# Proof of concept for a single-dose Group B *Streptococcus* vaccine based on capsular polysaccharide conjugated to Qβ virus-like particles

**DOI:** 10.1038/s41541-023-00744-5

**Published:** 2023-10-06

**Authors:** Filippo Carboni, Roberta Cozzi, Giacomo Romagnoli, Giovanna Tuscano, Cristiana Balocchi, Giada Buffi, Margherita Bodini, Cecilia Brettoni, Fabiola Giusti, Sara Marchi, Giulia Brogioni, Barbara Brogioni, Paolo Cinelli, Luigia Cappelli, Chiara Nocciolini, Silvia Senesi, Claudia Facciotti, Elisabetta Frigimelica, Monica Fabbrini, Daniela Stranges, Silvana Savino, Domenico Maione, Roberto Adamo, Benjamin Wizel, Immaculada Margarit, Maria Rosaria Romano

**Affiliations:** 1 GSK, Siena, Italy; 2 GSK, Rockville, MD USA

**Keywords:** Bacterial infection, Conjugate vaccines

## Abstract

A maternal vaccine to protect neonates against Group B *Streptococcus* invasive infection is an unmet medical need. Such a vaccine should ideally be offered during the third trimester of pregnancy and induce strong immune responses after a single dose to maximize the time for placental transfer of protective antibodies. A key target antigen is the capsular polysaccharide, an anti-phagocytic virulence factor that elicits protective antibodies when conjugated to carrier proteins. The most prevalent polysaccharide serotypes conjugated to tetanus or diphtheria toxoids have been tested in humans as monovalent and multivalent formulations, showing excellent safety profiles and immunogenicity. However, responses were suboptimal in unprimed individuals after a single shot, the ideal schedule for vaccination during the third trimester of pregnancy. In the present study, we obtained and optimized self-assembling virus-like particles conjugated to Group B *Streptococcus* capsular polysaccharides. The resulting glyco-nanoparticles elicited strong immune responses in mice already after one immunization, providing pre-clinical proof of concept for a single-dose vaccine.

## Introduction

*Streptococcus agalactiae* (Group B *Streptococcus*, GBS) has adapted to human entero-genital environments and colonizes 15–30% of women^1^. During pregnancy, these bacteria can reach the fetus and cause stillbirth or preterm delivery^2^. Both pre-partum and peripartum infections can result in severe neonatal bacteremia, sepsis, and/or meningitis^3^. The risk of Early-Onset GBS disease in the first week of life has been reduced by intrapartum antibiotic prophylaxis^4^. However, this treatment does not protect against GBS acquisition by the fetus during pregnancy or Late-Onset GBS infection occurring between the second week and first three months^5^, and it is difficult to implement in poor-resourced countries^6^. A maternal vaccine against neonatal GBS infection is therefore an urgent medical need.

GBS is surrounded by a thick sialic-acid-rich capsular polysaccharide (PS), which is a major virulence factor comprising ten capsular PS serotypes^7,8^. Each PS when conjugated to carrier proteins and administered to female mice prior to mating, elicited serotype-specific functional antibodies that were efficiently transferred to pups and mediated their protection against GBS lethal challenge^9^. In humans, PS serotype-specific maternal antibody titers inversely correlated with the risk for infection in infants^10^, providing the basis to develop a PS-based maternal vaccine for protecting neonates^11^. Interestingly, anti-PS IgG titers in mouse and human sera strongly correlated with opsonophagocytic killing titers, as well as with the levels of protection against GBS infection in a passive immunization-neonatal challenge mouse model^12^.

Maternal vaccines are offered during the third trimester of pregnancy to maximize their safety, which leaves a short temporal window to administer multiple doses. Therefore, a single-dose formulation would be optimal for this population. GBS vaccines containing one or multiple PS serotypes conjugated to Cross Reactive Material (CRM, a non-toxic mutant of Diphtheria Toxin) or Tetanus Toxoid (TT) have demonstrated to be safe in human studies^13–17^. However, one challenge encountered was the wide range of measured anti-PS antibody titers after administration of a single dose of vaccine. Indeed, while PS-specific IgG responses were very high in individuals with pre-immune titers above the Limit of Quantification (LOQ), and therefore presumably primed by GBS colonization, those presenting baseline IgG titers below the LOQ showed much lower responses. Antibody responses in these unprimed subjects could not be improved using adjuvanted formulations or by two vaccine doses administered after one or two month intervals^18^, while a second dose offered more than 1 year apart strongly boosted PS-specific responses^13,19^. These observations suggested that GBS PS conjugates were ideal for boosting but might be sub-optimal priming vaccines. A need for a second dose has also been observed for vaccines targeting GBS proteins both in preclinical models^20,21^ and in humans^22^.

In the present study we investigated the potential of an alternative vaccine presentation by conjugating PS to Protein Nanoparticles (NPs) and Virus-Like Particles (VLPs). NPs and VLPs can be potent delivery systems for protein antigens due to their large size and dense antigen display that enhance immune-cell uptake and activation^23^. Several vaccines based on NPs and VLPs have been developed for delivery of viral peptide and protein antigens^24–30^.

NPs and VLPs are also receiving growing interest as carriers of polypeptide antigens for developing vaccines against bacterial pathogens^31–34^. However, they have been less studied as carriers of saccharide antigens^35,36^. Here we compared different self-assembling NPs and VLPs as scaffolds for GBS PS antigens to improve their immunogenicity after a single vaccine dose.

## Results

### A single dose of GBS PSII conjugated to Qβ VLPs elicits IgG and functional antibody responses comparable to two doses of PSII-CRM conjugates

Similar to humans who are unprimed and present a low immune response to GBS PS conjugated to proteins (TT or CRM), at least two booster doses are required to elicit peak IgG responses in mice, rabbits^9,37^, and non-human primates^38^. Therefore, we assessed PS conjugated to self-assembling NPs and VLPs for possible enhanced immune responses in a mouse immunization model. The type II capsular polysaccharide (PSII), one of the six most abundant serotypes (Ia, Ib, II, III, IV, and V), was conjugated to NPs varying in subunit number and size, or to CRM as a benchmark. The self-assembling NPs Ferritin^39^ and mI3^40^ as well as bacteriophage Qβ coat protein VLP, with diameters of 10, 18, and 30 nanometers, respectively, were selected and expressed as recombinant proteins from genetically engineered *E. coli*. These nanoparticle carriers and CRM were conjugated to PSII, after random partial oxidation with sodium periodate, via reductive amination of ε-amine groups of lysines^8^. The resulting PSII conjugates were purified by Tangential Flow Filtration and characterized for protein and saccharide content and tested for immunogenicity in mice. The integrity of the nanoparticles before and after conjugation was confirmed by size-exclusion High Performance Liquid Chromatography (HPLC), Differential Light Scattering (DLS), and negative-stain Transmission Electron Microscopy (TEM) (Supplementary Table 1 and Supplementary Fig. 1).

In a first immunization experiment, CD-1 mice (10 per group) received two doses, three weeks apart, of the PSII-NP/VLP or PSII-CRM conjugates (0.5 µg of PSII/dose), adjuvanted with aluminum hydroxide (Alum). Sera were collected three weeks after the first dose and two weeks after the second dose for measuring IgG titers by Luminex, using randomly biotinylated PSII as the coupling reagent. In addition, pooled sera were evaluated in an Opsonophagocytic Killing Assay (OPKA) that mimics the in vivo killing of GBS by effector cells in the presence of complement. In previous studies, increasing OPKA titers strongly correlated with protection after direct immunization in a neonatal mouse challenge model and after passive transfer of maternally immunized human cord serum to newborn mice^12^.

In all mice, preimmune IgG and OPKA titers were below the limits of quantification (<20 for Luminex assay and <30 for OPK assay). As shown in Table 1, after two immunizations (post-2), all the conjugates elicited strong anti-PSII IgG and OPKA responses, demonstrating that the tested nanoparticles were good carriers for the capsular polysaccharide. However, PSII-Qβ elicited IgG and OPKA titers greater than the other NP- or the CRM-conjugates. Intriguingly, a single dose of the PSII-Qβ conjugate elicited IgG and OPKA titers comparable to those elicited by two doses of PSII-CRM or the other PSII-NP conjugates, while after one immunization (post-1) responses in the other groups were null or very low.

**Table 1:** Anti-PSII IgG and OPKA titers in pooled serum samples from mice after one and two doses of the indicated PSII conjugates formulated with Alum where IgG titers are reported as relative Luminex units per mL (RLU/mL) and OPKA titers as serum dilutions mediating 50% bacterial killing.

Conjugate	Post-1		Post-2	
	Anti PSII IgG titer	OPKA titer	Anti PSII IgG titer	OPKA titer
PSII-CRM	587	<30	8774	432
PSII-ferritin	1295	53	8341	610
PSII-mI3	542	<30	4088	192
PSII-Qβ	4160	746	15,721	2932

### A single dose of PSII-Qβ elicits persistent IgG and OPKA responses

A second immunization experiment was conducted to confirm the results obtained with PSII-Qβ and to assess the persistence of the immune responses after a single dose. Mice were immunized twice with PSII-CRM on days 1 and 21, or once with PSII-Qβ, and sera were collected at days 21, 42, 64, 99, and 134 post-immunization for analysis of anti-PSII IgG (Luminex, individual mouse sera) and OPKA titers (pooled sera).

**Fig. 1:**
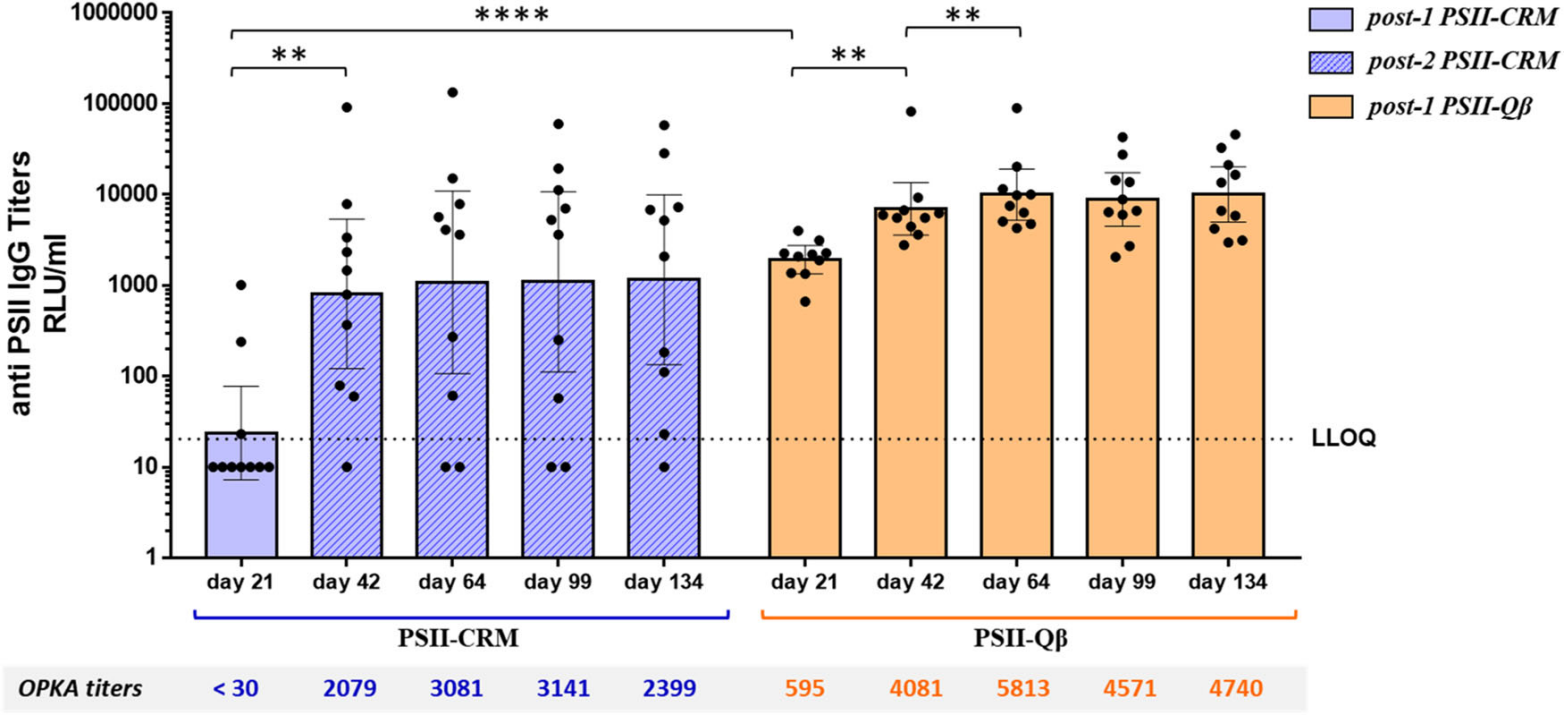
Persitence of anti-PSII IgG titers in animals vaccinated with two doses of PSII-CRM and one dose of PSII-Qβ conjugates. Serum samples were collected from groups of 10 immunized mice at times after one dose of PSII-Qβ or after one (day 21) or two doses of PSII-CRM conjugates. The geometric mean titer (RLU/mL) is indicated by the bars, individual mice are indicated by the dots, and the 95% Confidence Interval is indicated by the whiskers. For GMT, non responder sera were assigned titers half of the LLOQ. The Wilcoxon signed-ranks test was used to compare titers of the same immunization group and the Mann–Whitney test to compare different immunization groups. **P < 0.01; ****P < 0.0001. OPKA titers from pooled serum samples or each immunization group are reported below the histograms.

As shown in Fig. 1, two doses of PSII-CRM and one dose of PSII-Qβ induced IgG and OPKA titers that persisted until the end of the experiment on day 134. By day 21, a single dose of PSII-Qβ already elicited remarkably high IgG titers, and they increased along with opsonophagocytic titers to peak on day 42 and persist out to day 134. Of note, responses were more homogeneous among animals receiving the PSII-Qβ conjugate compared to those immunized with CRM conjugate. A further separate experiment revealed that the responses to a single dose of PSII-CRM did not increase after 42 days but remained as low as on day 21, requiring a booster dose to elicit functional activity in most animals (Supplementary Fig. 2).

### An additional GBS PS type conjugated to Qβ VLP induces robust IgG and OPKA responses after a single dose

**Fig. 2:**
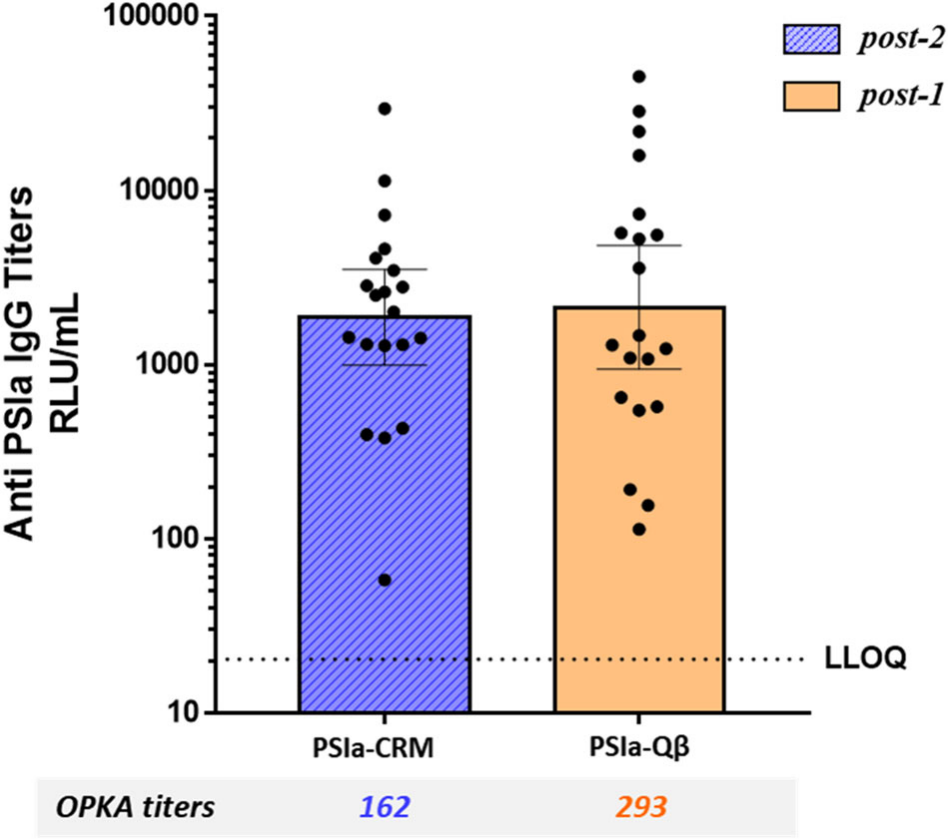
Antibody responses in mice receiving GBS PSIa-CRM or -Qβ conjugates. PSIa IgG titers in serum samples collected from mice (20 per group) receiving two doses of PSIa-CRM or one dose of PSIa-Qβ. The geometric mean titer (RLU/mL) is indicated by the bars, individual mice are indicated by the dots, and the 95% confidence interval is indicated by the whiskers. IgG titers between the two groups were compared by Mann–Whitney test. OPKA titers from pooled serum samples samples of each immunization group are reported below the barchart.

To assess whether a different GBS saccharide antigen conjugated to Qβ VLP might also elicit a strong immune response after a single dose, we used the same chemistry to conjugate capsular polysaccharide Ia (PSIa) to Qβ or CRM as a reference (Supplementary Table 1). Mice received a single shot of PSIa-Qβ or two shots of PSIa-CRM (0.5 µg/mouse adjuvanted with Alum), and their anti-PSIa responses were compared. While PSIa-CRM did not show any post-1 titer, the antibody titers after one dose of the PSIa-Qβ conjugate or two doses of PSIa-CRM conjugate were not statistically different, and OPK titers of pooled sera were also comparable, as shown in Fig. 2. Thus, the Qβ VLP proved an optimal carrier for a single-dose GBS vaccine with two different PS types.

### Plasmid genetic manipulation allowed to obtain a Qβ carrier nanoparticle with highly homogenous entrapped-RNA

We tested post-2 immune responses to PSII-Qβ with and without Alum and no significant difference was found (Supplementary Fig. 3). This observation was not unexpected, since bacterial host RNA randomly trapped in Qβ VLPs^41^ during assembly can activate endosomal TLR7 and 8, stimulating innate immunity that contributes to the immunogenicity of Qβ VLP vaccines^42^. Indeed, the Qβ infectious phage particle packages its single-stranded RNA genome by virtue of a high-affinity interaction between a hairpin structure and interior-facing residues of the Qβ coat protein^43,44^, but can also entrap RNA derived from its host cell^41^. Therefore, a tailored analytical characterization was deemed essential to ensure consistency between different vaccine batches and their induced immune responses.

RNA samples extracted from multiple Qβ batches were analyzed in terms of total amount and size distribution. RNA content was comparable between the different Qβ preparations, ranging from 20 to 30% of the total VLP mass, while microfluidic capillary electrophoresis showed non-homogenous profiles with large variations in the relative amount of RNA of different lengths (Supplementary Fig. 4a).

**Fig. 3:**
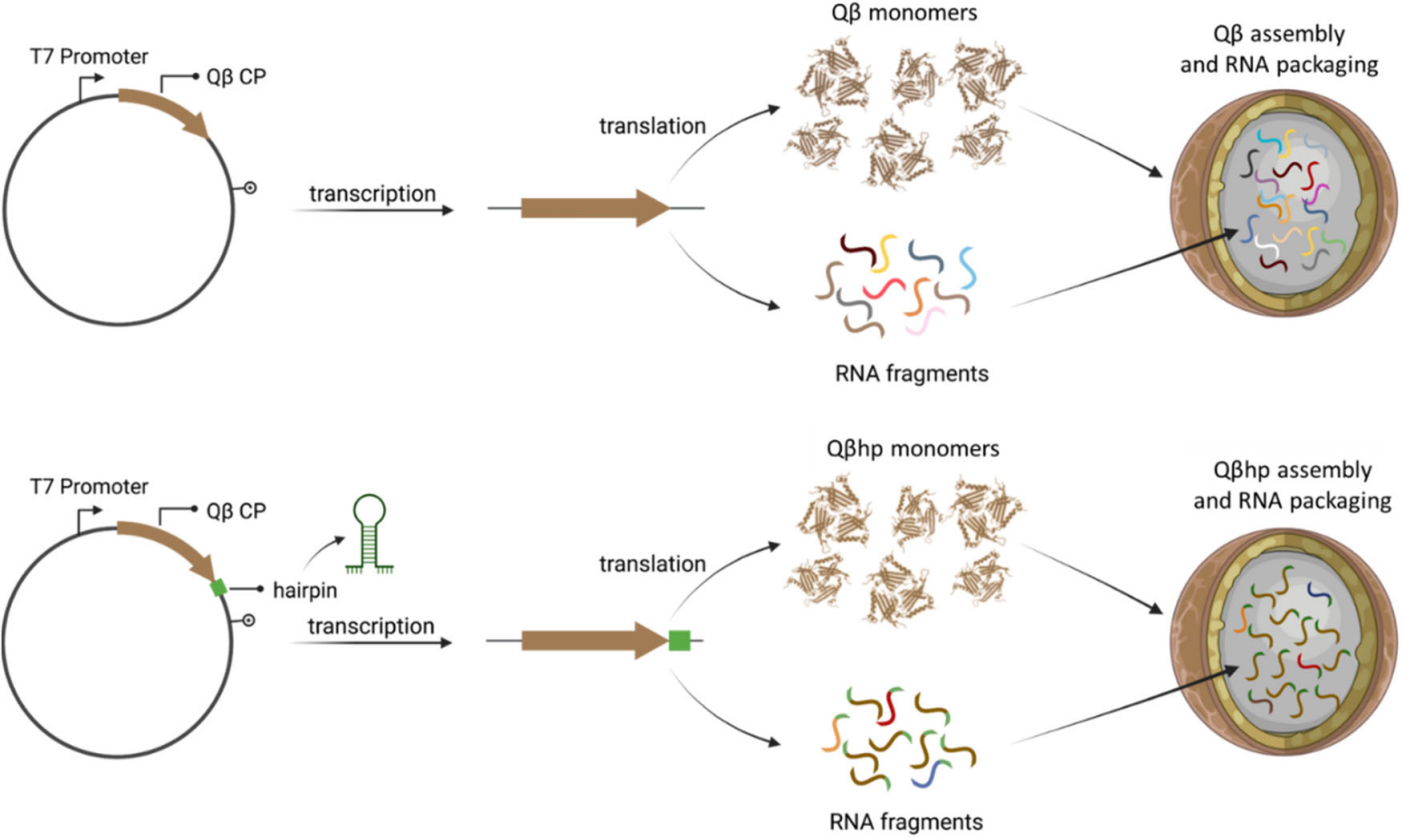
Genetic strategy used for the generation of Qβ and Qβhp VLPs. Top panel: expression of Qβ VLPs was achieved by cloning the Qβ coat protein open-reading frame under the T7 promoter; monomers self-assemble drandomly incorporating *E.coli* RNA. Bottom panel: for Qβhp VLPs expression, a hairpin structure was inserted immediately downstream of the stop codon of the gene encoding the Qβ coat protein; monomers self-assembled incorporating mainly Qβhp RNA.

To obtain nanoparticles containing more homogeneous RNA, we modified the *E. coli* expression plasmid by inserting a hairpin structure derived from the Qβ genome immediately downstream of the stop codon of the gene encoding the Qβ coat protein^45^. The devised genetic strategy is described in Fig. 3. We named the resulting VLPs, Qβhp^46^, and characterized their entrapped RNA versus the RNA in the wild-type Qβ. The two VLPs contained similar quantities of RNA, but the size distribution of the RNA in the Qβhp was much more homogeneous in two independent batches, with a major peak corresponding to the Qβ coat protein mRNA (ca. 800 nt, Supplementary Fig. 4b).

**Fig. 4:**
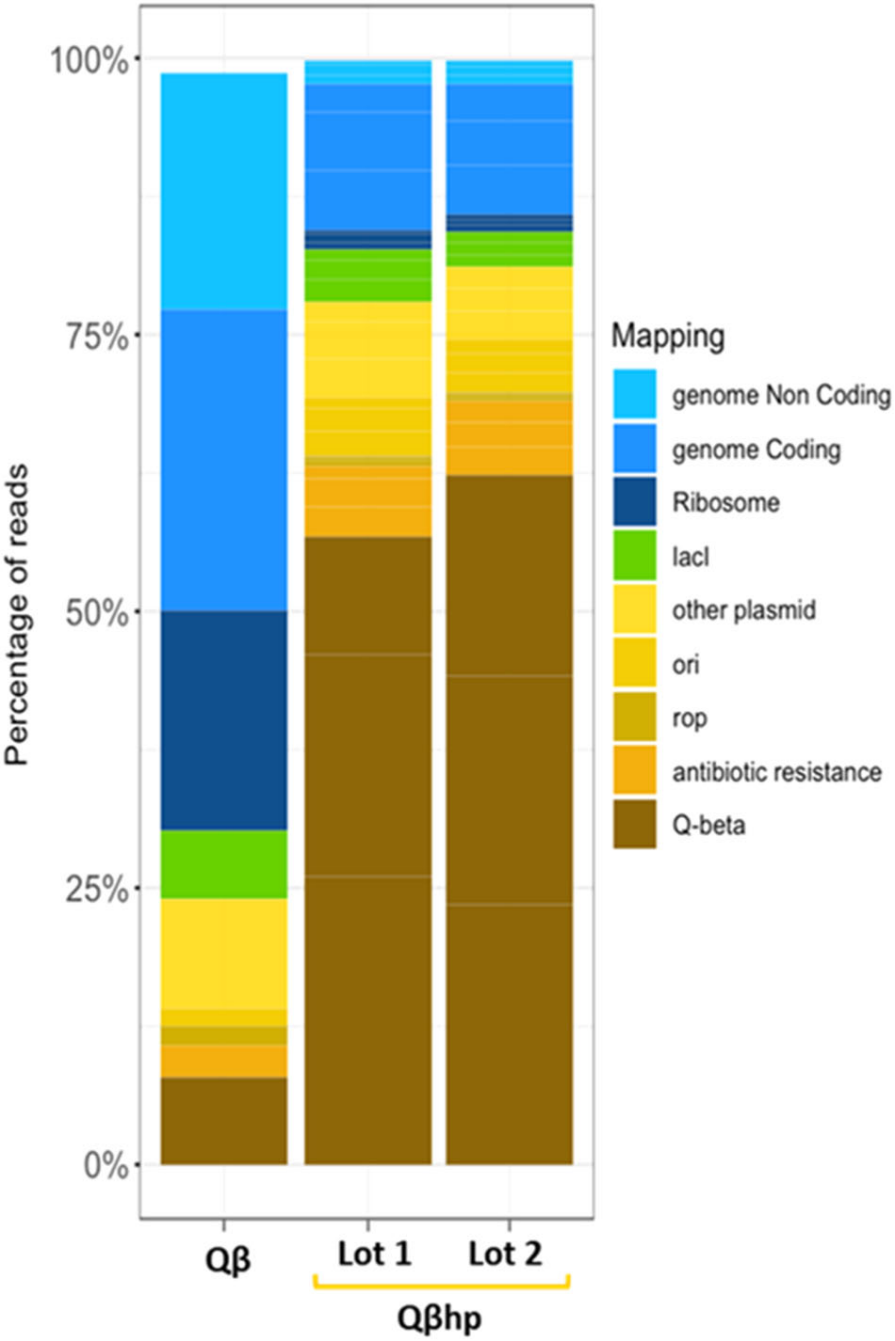
Sequencing analysis of RNA extracted from Qβ and Qβhp. Quantification of the different categories of RNA-seq reads is indicated as a percentage of the total number of reads in samples from 1 lot of Qβ (first lane) and 2 lots of Qβhp (second and third lanes). In the bar plot, shades of blue are used for reads mapping to the *E. coli* BL21DE3 genomic sequence and shades of yellow and brown for reads mapping to the pET24b+ plasmid. lacI is reported in green as it is present in two copies on the genome and in one copy on the plasmid.

Next, we analyzed the Qβhp and Qβ VLPs for the sequences of their entrapped RNA. As shown in Fig. 4, Qβ trapped RNA was mostly from *E. coli*, with a majority being rRNA and ribosomal protein transcripts. Conversely, the RNA from Qβhp VLPs mapped mostly to the expression plasmid, with ~55% encoding Qβ coat protein (that further increased when the bacteria were grown in chemically defined media), ~25% encoding other plasmid genes, and <20% mapping to the *E. coli* genome. Aside from the RNA quality, Qβ and Qβhp VLPs showed no difference in purity, size, or final yield (>90% purity by SEC-HPLC, 16 nm radius by DLS, 2–3 mg/g of biomass for both).

### Entrapped RNA and multivalency both contribute to immunogenicity of PSII-Qβ conjugates

**Fig. 5:**
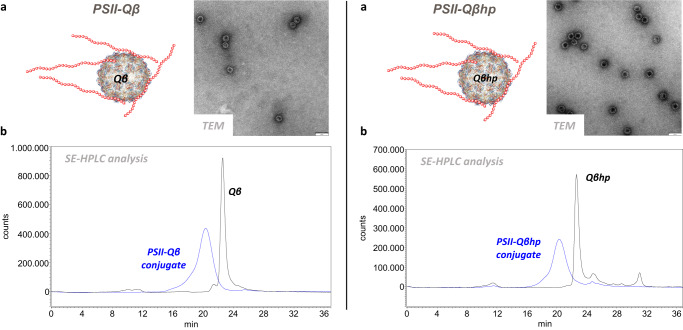
Analytical characterization of PSII-Qβ (left) and PSII-Qβhp conjugates (right). **a** Transmission electron microscopy (TEM) in negative staining, and **b** Size-exclusion High Performance Liquid Chromatography (SE-HPLC).

**Fig. 6:**
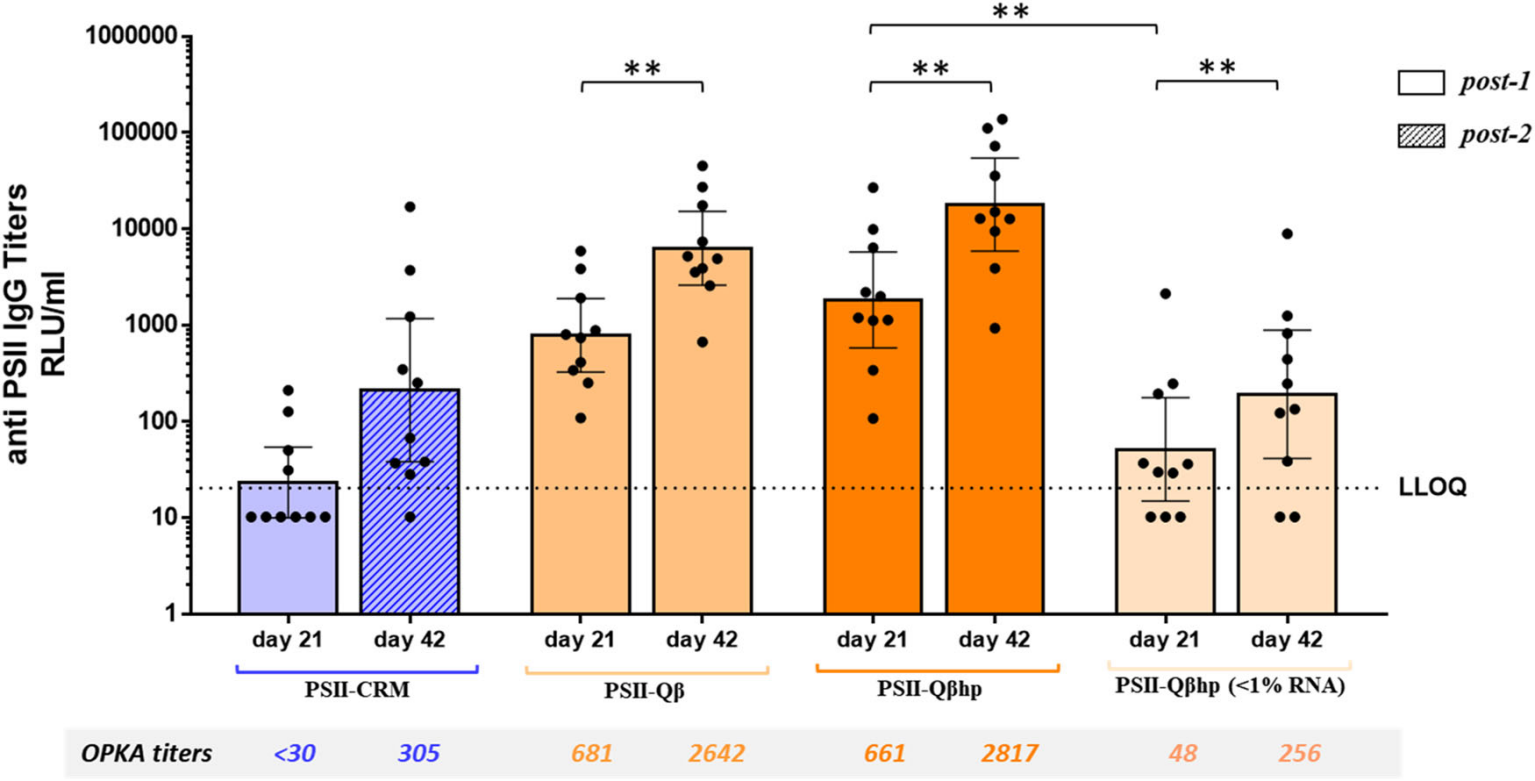
Immune responses in mice receiving PSII conjugated to different Qβ VLPs. Mice (10 per group) received one (full bars) or two doses (patterned bar) of PSII-CRM, PSII-Qβ, PSII-Qβhp, or RNAse-treated PSII-Qβhp. The geometric mean IgG titer (RLU/mL) is indicated by the bars, individual mice are indicated by the dots, and the 95% Confidence Interval is indicated by the whiskers. For GMT, non responder sera were assigned titers half of the LLOQ. The Wilcoxon signed-ranks test was used to compare titers at day 21 and day 42 of the same immunization group of the same immunization group and the Kruskal–Wallis and Dunn multiple comparisons test was used to compare different immunization groups. **P < 0.01. The corresponding OPKA titers from pools of serum samples are reported below the barchart.

To assess the carrier properties of the new construct, Qβhp VLPs were conjugated to PSII using the same conjugation procedure as above and analyzed by TEM and by size-exclusion HPLC, showing comparable physico-chemical features to PSII-Qβ (Fig. 5). Groups of 10 mice received a single dose of PSII-Qβhp or PSII-Qβ, or two doses of PSII-CRM as reference. As shown in Fig. 6, there was no statistical difference in IgG and OPKA titers elicited by one dose of PSII-Qβhp or PSII-Qβ, or two doses of PSII-CRM, and antibodies elicited by both Qβ and Qβhp conjugates further increased over time.

In the same experiment, we investigated the adjuvant effect of the Qβhp trapped RNA by immunizing a further group of animals with a PSII-Qβhp conjugate containing <1% of RNA after RNAse treatment. In mice receiving RNAse-treated PSII-Qβhp, the anti-PSII IgG and OPKA titers on day 21 and 42 were more than one log lower than those elicited by the untreated PSII-Qβhp, confirming the adjuvant role of encapsidated RNA.

After the single dose of RNAse-treated PSII-Qβhp, the immune responses increased from day 21 to 42, reaching levels similar to those elicited by two doses of PSII-CRM (although still lower than PSII-Qβhp). This result was presumably due to the multivalent display of PS by the VLP, because a single dose of the classical PSII-CRM conjugate did not cause the immune response to increase over time (Supplementary Fig. 2).

### A single dose of PSII-Qβhp VLPs elicits anti-PSII IgG and OPKA titers greater than those elicited by a single dose of PSII-CRM formulated with different adjuvants

**Fig. 7:**
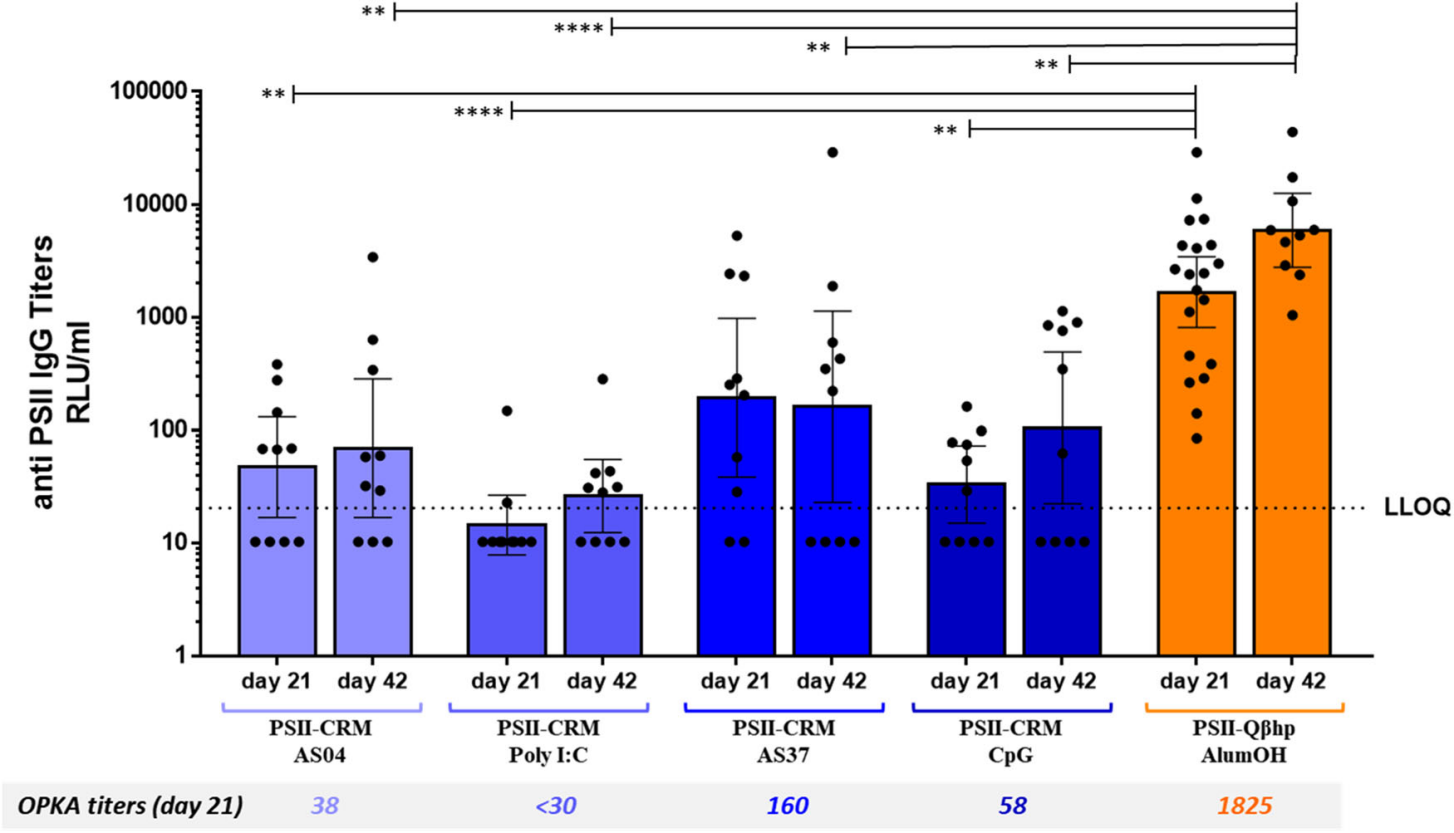
Immune responses in mice receiving PSII-CRM with different adjuvants. Mice (10 per group) received a single dose of PSII-CRM formulated with AS04, Poly I:C, AS37 and CpG or PSII-Qβhp formulated with Aluminum hydroxide. The geometric mean IgG titer (RLU/mL) at day 21 and 42 is indicated by the bars, individual mice are indicated by the dots, and the 95% confidence interval is indicated by the whiskers. For GMT, non responder sera were assigned titers half of the LLOQ. The Kruskal–Wallis and Dunn multiple comparisons test was used to compare the group receiving PSII-Qβhp with all PSII-CRM immunization groups. **P < 0.01; ****P < 0.0001. The OPKA titers from pools of serum samples at day 21 are reported below the barchart.

Next, we assessed the possibility that vaccination with PSII-CRM formulated with adjuvants targeting diverse Toll-like Receptors (TLR) and immune pathways might elicit, after a single-dose, anti-PSII IgG and OPKA titers as high or higher than those elicited by a single dose of PSII-Qβhp. We injected mice with a single dose of PSII-Qβhp formulated with Alum, or PSII-CRM formulated with AS04, Poly I:C, AS37, or class B CpG, which are agonists of TLR4, TLR3, TLR7, and TLR9, respectively. As shown in Fig. 7, the adjuvant with PSII-CRM that elicited the highest anti-PSII IgG and OPKA titers was AS37, a TLR7 agonist. However, titers were not as high as those elicited by PSII-Qβhp. Thus, the adjuvancy provided by entrapped RNA within the Qβ VLP was superior to that of the adjuvants that were simply co-administered.

## Discussion

Self-assembling NPs are offering new avenues as vaccine delivery vehicles for improving immune responses to subunit vaccines. As large oligomeric structures, NPs can be engineered to display multiple antigens in a single particle, facilitating their uptake by antigen-presenting cells for efficient recognition and presentation to specific surface receptors of immune cells^23,44,47,48^. Several vaccines based on NPs and VLPs have been developed for delivery of viral peptide and protein antigens from flu^24,25^, HIV^26^, RSV^27^, and SARS CoV-2^28–30^. Surface decoration of nanoparticles with protein antigens has also shown promise for application to bacterial vaccines^31–34^. Recent studies have investigated NPs and VLPs as carriers of bacterial saccharide antigens with the aim of improving T-cell help to anti-glycan B cells. Polonskaya and colleagues pioneered the display of *S. pneumoniae* synthetic tetra-saccharides of serotypes 3 and 14 on the surface of VLPs, resulting in strong and persistent post-2 and post-3 serotype-specific IgG responses that protected mice against infectious challenge^35^. More recently, a modular biosynthetic approach to produce nanoconjugate vaccines using the SpyTag/SpyCatcher system was applied for the direct coupling of *Shigella flexneri* native polysaccharides to AP205 and Qβ VLPs. The authors demonstrated an increase in Tfh and germinal center B cells in draining lymph nodes, compared to classical protein carrier conjugates, and an efficient adaptive immune response and prophylaxis against bacterial challenge^36^.

We investigated the possibility to develop a GBS vaccine for a single administration during the third trimester of pregnancy, leveraging NPs capability to elicit potent immune responses. Capsular polysaccharide II was chemically conjugated to Ferritin and mI3 NPs and Qβ VLPs. After vaccinating mice with two doses, all of these nanoconjugates elicited PSII-specific antibody functional responses higher than the reference CRM conjugate, confirming that self-assembling NPs are potent delivery systems for polysaccharide antigens that can be exploited as an alternative to classical and widely used carrier proteins^49^. The Qβ conjugate elicited post-1 responses higher than the other nanoconjugates and like those obtained after two doses of PSII-CRM, confirming Qβ VLP as a suitable system to enhance responses against poorly immunogenic saccharide targets. Further, IgG and OPKA titers induced by the PSII-Qβ conjugate reached a maximum on day 42 and persisted up to day 134. The immune-enhancement of the Qβ carrier was further confirmed with capsular polysaccharide Ia (PSIa), suggesting the same approach could be applicable and tested for the other CPS.

Our data are also in line with previous literature showing that Qβ-VLPs can enhance immune responses to other weak or non-immunogenic antigens like small peptides and glycopeptides, as demonstrated for human vaccines against nicotine dependence, hypertension, allergies, diabetes, cancer, and Alzheimer’s disease and are safe and highly immunogenic^43,50–54^. Further, this study confirm the self-adjuvant effect of VLPs associated with the presence of entrapped RNA, which acts as a scaffold for the self-assembly of prophage coat protein monomers, and activates the innate immune system via TLR7 and 8^55^.

Encapsulated RNA represents for about 20–30% of the Qβ VLP mass and mainly consists of hairpin-rich molecules derived from the prophage or encoded in the bacterial host genome^56,57^, resulting in fragments of highly variable size and composition. Rhee and colleagues demonstrated that RNA encoding the green fluorescent protein (GFP) could be delivered into Qβ VLPs by adding a hairpin sequence to the end of its coding gene^58^. To achieve a more reproducible RNA profile of our VLPs, we designed an *E. coli* strain where the hairpin sequence was added to the plasmid-encoded Qβ coat protein gene, resulting in a VLP containing RNA with a more homogeneous profile, mainly represented by a single major peak encoding the Qβ coat protein, the new Qβhp^45,59,60^.

Like its precursor, PSII-Qβ, the PSII-Qβhp conjugate induced high IgG and OPKA titers after a single vaccine dose. To confirm the effect of the VLP-entrapped RNA as immune potentiator and to better understand if the VLP size and ability to present multiple copies of the target antigen on its surface could also play a role in the observed enhanced immune responses, RNA was almost completely removed from the PSII-Qβhp conjugate by RNAse treatment. This caused a strong decrease in anti-PSII immune responses; however, 42 days after a single dose of the RNA-depleted VLP conjugate, antibody levels were like those in animals receiving two doses of PSII-CRM. We concluded that the entrapped RNA and multivalent antigen presentation of the Qβ VLP both contribute to achieve optimal immune response. To support this hypothesis, we compared PSII-Qβhp with the PSII-CRM conjugate formulated with AS04, Poly I:C, AS37, or class B CpG (agonists of TLR4, TLR3, TLR7, and TLR 9, respectively)^61^. A single dose of PSII-CRM co-administered with these adjuvants did not induce IgG and OPKA responses comparable to those observed with PSII-Qβ, suggesting that the presentation of the antigen and the immune potentiator in a single VLP offers an advantage compared to administering them as separate components in the same formulation.

The immune-enhancing effect of Qβ as antigen carrier has been appreciated beyond rodent species. Phares and colleagues compared the immunogenicity of malaria candidate vaccines containing a recombinant circumsporozoite protein or derived polypeptides, either in soluble form or conjugated to Qβ, both in mice and in non-human primates. Interestingly, they found higher antibody titers after a single dose of the VLP conjugates than after a single dose of the unconjugated antigens in both species^62^.

In conclusion, Qβ VLPs offer promise as carriers for the delivery of saccharide antigens against group B *Streptococcus* and beyond, and can be particularly attractive for the development of single-dose vaccines.

## Methods

### Expression and purification of nanoparticles

The genes encoding for ferritin, mI3 and Qβ were synthesized as DNA strings by GeneArt (Thermo Fisher Scientific) optimizing the codon usage for expression in the *E. coli*. The genes were cloned into pET15b+TEV and pET21b+ (Merck-Sigma) PCR-amplified vectors using the Infusion cloning kit (Takara) following manufacturer instructions^33,63^. The plasmid pET24b+ encoding the Qβhp coat protein was purchased by GeneArt (Thermo Fisher Scientific).

All recombinant proteins were expressed in *E. coli* BL21(DE3) (New England Biolabs). For ml3 expression, cells were grown in HTMC medium (Glycerol 15 g/L; Yeast Extract 30 g/L; MgSO_4_ ×7H_2_O 0.5 g/L; KH_2_PO_4_ 5 g/L; K_2_HPO_4_ 20 g/L; KOH 1 M to pH final 7.35 ± 0.1) supplemented with Kanamycin, under shaking at 20 °C for 16 h followed by induction with 1 mM IPTG for 24 h. Qβ and Qβhp particles were produced using a 5 L bioreactor (Applikon) set at controlled temperature of 32 °C, pH 6.50 ± 0.1 (by the addition of 4 M NaOH and 2 M H_3_PO_4_ when needed), 400–800 RPM, air flow rate between 1–2 VVM and 30%–100% dissolved oxygen (by automatic enrichment of the inlet air stream with Air and RPM). For the production of Qβ and Qβhp lot 1 host *E. coli* cells were grown in HTMC; when an OD_590_ value of 5 was reached, Induction Solution (Glycerol 30 g/l; CaCl_2_ 0.12 g/L; MgSO_4_ 0.15 g/L; IPTG 1 mM) was added to the culture and the biomass recovered after 5 h. For Qβhp lot 2 production, bacteria were cultured in M9 modified medium (Glycerol 30 g/L; NH_4_Cl 3 g/L; KH_2_PO_4_ 14 g/L; K_2_HPO_4_ 6 g/L; MgSO_4_ 0.24 g/L; Riboflavin 0.002 g/L; Thiamine 0.002 g/L; Pyridoxine hydrochloride 0.002 g/L; Nicotinamide 0.002 g/L; FeSO_4_ 1.53 mg/L; MnCl_2_ 2.64 mg/L; CuSO_4_ 1.98 mg/L; ZnSO_4_ 0.34 mg/L; Na_2_MoO_4_ 0.6 mg/L); when an OD_590_ value of 3 was reached, induction solution (Glycerol 30 g/l; CaCl_2_ 0.12 g/L; MgSO_4_ 0.15 g/L; (NH_4_)_2_SO_4_ 6 g/L; FeSO_4_ 9.18 mg/L; MnCl_2_ 15.84 mg/L; CuSO_4_ 6.78 mg/L; ZnSO_4_ 11.88 mg/L; Na_2_MoO_4_ 2.04 mg/L; IPTG 1 mM) was added and the biomass recovered.

For mI3 purification, bacterial cells were chemically lysed with CelLytic Express (SIGMA), followed by several steps of purification, in sequence: Affinity Chromatography Hi Trap HP, Ni^2+^ (Cytiva), Size Exclusion Chromatography Sephacryl S300 (Cytiva), Endotrap Column RED (IlexLife) and Hydroxyapatite column CHT (BIO-RAD) for endotoxin removal. Pooled fractions were stored in 100 mM NaH_2_PO_4_ pH7,6 at −20 °C until further use. For Qβ and Qβhp purification, bacterial cells were subjected to mechanical lysis, and the supernatant flocculated with Polyethyleneimine (PEI). After centrifugation, the flocculate containing Qβ or Qβhp was purified by Ionic Exchange Chromatography (Cytiva), followed by Size Exclusion Chromatography Sephacryl S500 (Cytiva) in PBS; the purified VLPs were stored at −20 °C until further use. Protein concentration was determined by Pierce™ Rapid Gold BCA Protein Assay Kit (Thermo Fisher Scientific, Illinois, USA) according to manufacturer’s instructions, using Bovine Serum Albumin (BSA) as standard.

### Analytical characterization of Qβ and Qβhp VLPs

Size exclusion chromatography was performed on a TSKgel G6000PW column (17 µm, 7.5 × 300 mm, Tosoh, Japan) using an Alliance Bio HPLC system (Waters, Massachussets, USA) equipped with a photodiode array detector and a multiple-wavelength fluorescence detector. Analysis was performed with an isocratic mobile phase of PBS, at 0.5 mL/min, at room temperature. A multi-angle light scattering (MALS) system was coupled downstream of the HPLC. MALS signals were detected by a Dawn Heleos – II detector and an Optilab T-rEX refractive index (RI) detector (Wyatt, Santa Barbara, CA). ASTRA 7.3.1 software was used for acquiring and analyzing UV, RI, and MALS data.

VLP Particle Size Distribution was assessed by DLS with the DynaPro PlateReader-II (Wyatt Technology, California, USA) using 80 µL of sample/well and 35 measurements/sample.

For Transmission Electron Microscopy analysis, 5 µL of samples (20 ng/µL) in PBS were loaded for 30 s on a glow discharger copper 200-square mesh grids (EMS). Blotted the excess, the grid was negatively stained using NanoW (Nanoprobes) for 30 s and let air dried. Samples were analyzed by a Tecnai G2 spirit and images acquired using a Tvips TemCam-F216 (EM-Menu software).

### Analysis of Qβ and Qβhp RNA content

One hundred µg of purified VLPs were lysed with 600 µL of Trizol at RT for 5 min and 600 µL of absolute ethanol were added to the solution. RNA was extracted by Pure link RNA Mini Kit according to the manufacturer’s instructions. Total RNA was quantified by Quant-iT™ RiboGreen® RNA Reagent and Kit (Molecular Probes®, Oregon, USA), using rRNA as standard. Native (untreated) and denaturated (by incubation with 4 M urea for 30 min at 95 °C) Qβ VLPs were diluted in TE buffer (10 mM Tris-HCl, EDTA, pH 7.5) and 100 µL of RiboGreen were added to 100 µL of sample; fluorescence intensity (Excitation/Emission 485 nm/530 nm) was measured (Infinite 200, Tecan). RNA total content was expressed as µg/mL or % RNA/Protein.

For capillary electrophoresis analysis, 20 mL of purified RNA samples were loaded onto LabCHip GX II (PerkinElmer) following manufacturer’s instructions. Each sample was analyzed in triplicate.

For sequencing analysis, three RNA libraries were prepared from each sample starting with 100 ng of RNA (quantified by Agilent Bioanalyzer RNA 6000 Nano Kit) following the Illumina protocol Stranded Total RNA Prep with Ribo-Zero Plus (Illumina, San Diego, CA). The ribosomal RNA depletion step was omitted. Samples were sequenced on Miseq Illumina platform PE 2 × 75 cycles. Reads quality was checked with fastQC version 0.11.9. As reference for mapping we used *E. coli* BL21(DE3) reference genome downloaded from the NCBI (GenBank accession: GCA_013167015.1) coupled with genetic sequences of pET 19ABXGYC and 20ADXZPC, downloaded from the vendor website. Reads were aligned with bowtie2 version 2.4.1 and counted using samtools version 1.9 and bedtools version 2.29.2.

### Conjugation of GBS polysaccharides to nanoparticles

GBS polysaccharide II or Ia were oxidized targeting 20% of sialic acid residues^37^. Samples were stirred with 0.1 M sodium periodate in 10 mM sodium phosphate buffer in the dark, for 2 h at room temperature. The mixture was purified by liquid chromatography using G-25 desalting columns. The oxidized polysaccharide was dissolved in a 100 mM sodium phosphate buffer at pH 7.2 and mixed to NPs at a final protein concentration of about 10 mg/mL. Sodium cyanoborohydride (1:1 w/w based on the amount of polysaccharide) was added to the solution. The reaction mixture was incubated at 37 °C for 3 days, quenched with sodium borohydride and purified by tangential flow filtration (Sartocon 100 kDa filter). The protein content was determined by BCA colorimetric assay. Finally, the extent of saccharide conjugation was evaluated by SDS-PAGE electrophoresis and SE-HPLC using a Sepax SRT-C 2000 column.

PSII-Qβ conjugates devoid of RNA were generated by incubation of the samples with an RNase Cocktail (Invitrogen), mixture of two highly purified ribonucleases, RNase A and RNase T1. After an overnight incubation at room temperature, RNAses and nucleic acids were eliminated by serial centrifugal filtration (100 kDa).

Saccharide quantification in the conjugates was performed by high-performance anion-exchange chromatography with pulsed amperometric detection (HPAEC-PAD). GBS PSII or PSIa standard samples at five increasing concentrations ranging between 0.5 and 10 μg/mL, were prepared to build the calibration curve. Test samples were diluted targeting the calibration curve midpoint, while free saccharide samples were analyzed undiluted after separation by solid-phase extraction (SPE) cartridges. The reference and conjugate samples were prepared in 4 M trifluoroacetic acid (Supelco), incubated at 100 °C for 3 h, dried under vacuum (SpeedVac Thermo), suspended in water, and filtered with 0.45 μm Phenex-NY (Phenomenex) filters. HPAEC-PAD analysis was performed with a Dionex ICS-6000 equipped with a CarboPac PA1 column (4 × 250 mm; Dionex) coupled with a PA1 guard to column (4 × 50 mm; Dionex). Samples were run at 1 mL/min, using an isocratic elution with 14 mM NaOH, followed by a washing step with 500 mM NaOH. The effluent was monitored using an electrochemical detector in the pulse amperometric mode, with a gold working electrode and an Ag/AgCl reference electrode. A quadruple-potential waveform for carbohydrates was applied. The resulting chromatographic data were processed using Chromeleon software 7.2 (Thermo Dionex), and sample concentrations were determined based on galactose content.

### In vivo experiments

Animal studies have been approved by the Italian Ministry of Health and were ethically reviewed by the local Animal Welfare Body. Animal studies were carried out at GSK facility in accordance with national/European legislation and the GSK Policies on the Care, Welfare and Treatment of Animals. Groups of 10 CD1 (ICR) 6–8 weeks old female mice, were immunized intramuscularly (IM) with GBS conjugates (0.5 μg of PS/dose) adjuvanted with Aluminum Hydroxide (2 mg/mL AlumOH in Histidine buffer pH6.5, 150 mM NaCl). In one of the experiments CRM conjugates were alternatively formulated with AS04 2 mg/mL (MPL based 200 μg/mL), AS37 (LHD153R based 200 μg/mL), Poly I:C (30 μg/dose) or CpG class B (30 μg/dose).

Formulates with Poly I:C (30 μg/dose) and CpG class B (30 μg/dose) were prepared in PBS pH7.4, those with AS04 and AS37 in Histidine buffer pH6.5, 150 mM NaCl. All formulations were controlled for pH, osmolality, presence of precipitates, antigen adsorption, endotoxin content and bioburden.

### Biotin-PS II immunoassay

The immune response to GBS PS conjugates was assessed by Luminex-based monoplex assay^64^ using Biotin-PS II or Ia (1 µg/mL) conjugated to 1.25 × 106 Radix High capacity (HC) Streptavidin magnetic beads. Eight steps of threefold dilutions of a standard serum (pool of sera from mice immunized with PSII-CRM used as reference) and samples were mixed with an equal volume of conjugated Biotin-PS beads (3000 beads/well) in a 96-well Greiner (Millipore Corporation, Billerica, MA) and incubated for 60 min at RT in the dark on a plate shaker at 600 rpm. After incubation, the beads were washed three times with 200 µl PBS. Each well was then loaded with 50 µl of a 1/100 dilution of PE-secondary anti-mouse IgG (Jackson Immunoresearch, 115-116-072) and the plates were incubated for 60 min with continued shaking. After washing, beads were suspended in 100 µL of PBS before the analysis with Bioplex 200. Data were acquired in real time by Bioplex Manager Software 6.2 (Bio-Rad Laboratories, Hercules, CA).

The median fluorescence intensity (MFI) was converted to RLU/mL by interpolation to the corresponding 5-PL Standard curve (8 dilution points). IgG concentrations (RLU/mL) were determined from the mean of two sample dilutions for which MFI signals were in the linear range of the standard curve.

### Opsonophagocytic killing assay (OPKA)

OPKA was conducted using differentiated HL-60 cells and the GBS strain DK21 (serotype II)^65^. OPK titers were expressed as the reciprocal serum dilution mediating 50% bacterial killing, estimated through piecewise linear interpolation of the dilution-killing OPK data. A fluorescent OPKA was adapted from literature^66,67^ to increase the throughput capacity to 384-wells and to avoid CFU counting by introducing a fluorescent dye (Alamar Blue) that allow to measure the viability endpoint of bacteria through a plate reader. The fluorescent OPKA used the same assay components and titration method. The lower limit of detection was 1:30 dilution and the assay coefficient of variation was ~30% for both assay formats.

### Supplementary information

Supplementary material

nr-reporting-summary_mr_v2

## Data Availability

The authors declare that data supporting the findings of this study are available within the paper and its supplementary information files. All relevant data are available from the authors.
