# Author Correction: Proof of concept for a single-dose Group B *Streptococcus* vaccine based on capsular polysaccharide conjugated to Qβ virus-like particles

**DOI:** 10.1038/s41541-024-00808-0

**Published:** 2024-01-25

**Authors:** Filippo Carboni, Roberta Cozzi, Giacomo Romagnoli, Giovanna Tuscano, Cristiana Balocchi, Giada Buffi, Margherita Bodini, Cecilia Brettoni, Fabiola Giusti, Sara Marchi, Giulia Brogioni, Barbara Brogioni, Paolo Cinelli, Luigia Cappelli, Chiara Nocciolini, Silvia Senesi, Claudia Facciotti, Elisabetta Frigimelica, Monica Fabbrini, Daniela Stranges, Silvana Savino, Domenico Maione, Roberto Adamo, Benjamin Wizel, Immaculada Margarit, Maria Rosaria Romano

**Affiliations:** 1 GSK, Siena, Italy; 2 GSK, Rockville, MD USA

**Keywords:** Bacterial infection, Conjugate vaccines

Correction to: *npj Vaccines* (2023) **8**:152; 10.1038/s41541-023-00744-5, published online 06 October 2023

In the original version of this Article, the author Roberto Adamo was incorrectly omitted. The HTML and PDF versions of the Article have been corrected.

