# Supplementary material for: Proof of concept for a single-dose Group B Streptococcus vaccine based on capsular polysaccharide conjugated to Qβ virus-like particles
Source: NPJ Vaccines. 2023 Oct 6;8:152. doi: 10.1038/s41541-023-00744-5 (PMC10558462; doi:10.1038/s41541-023-00744-5)
Supplement: Supplementary file 1 — Supplementary material [file 41541_2023_744_MOESM1_ESM.pdf]

SUPPLEMENTARY MATERIAL

Supplementary Table 1. Carrier proteins size and conjugates saccharide/protein ratio.

Conjugate	Protein monomer MW (kDa)	Nanoparticle subunits	NP diameter (nm)	Saccharide/Protein ratio (w/w)
PSII-CRM	58	-	-	1.4
PSII-ferritin	20	12	10	5.3
PSII-mI3	24	60	18	1.0
PSII-Qβ	15	180	30	0.1
PSII-Qβhp	15	180	30	0.1
PSII-Qβhp (<1% RNA)	15	180	30	0.1
PSIa-CRM	58	-	-	2.1
PSIa-Qβ	15	180	30	0.4

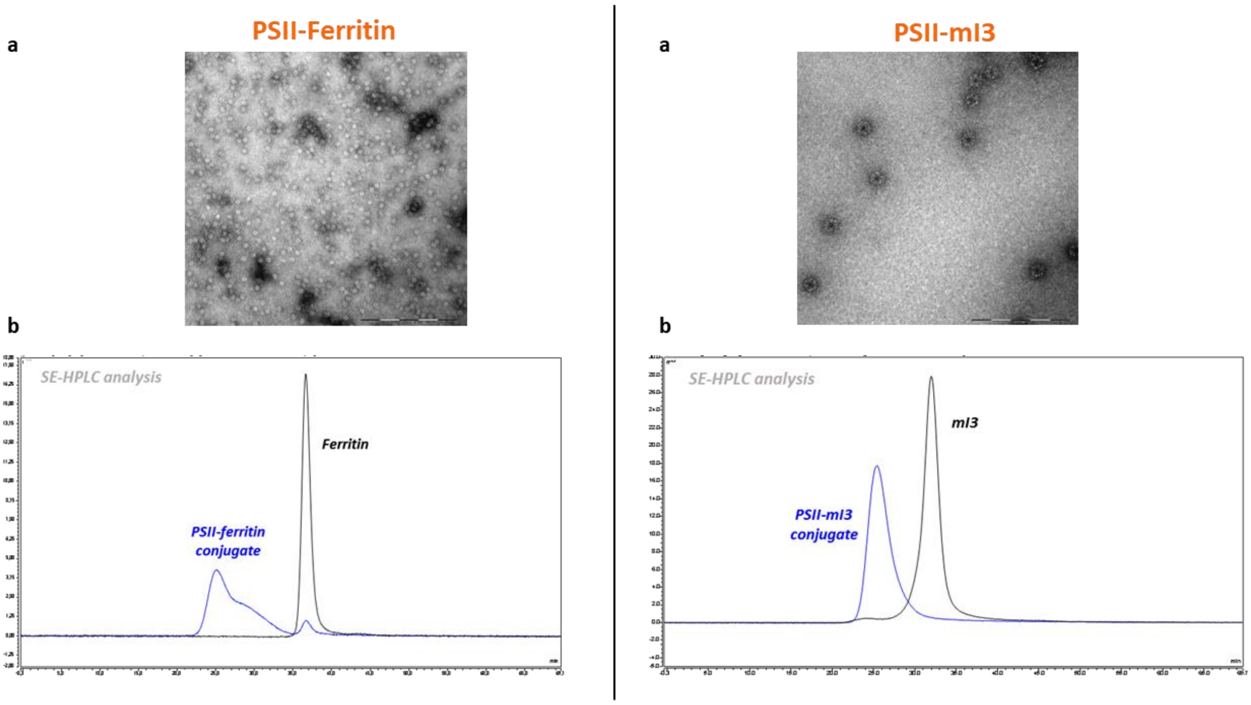

Supplementary Figure 1. Analytical characterization of PSII-ferritin (left) and PSII-mI3 (right).

(***a***) Transmission electron microscopy (TEM) in negative staining, (***b***) Size-exclusion High Performance Liquid Chromatography (SE-HPLC).

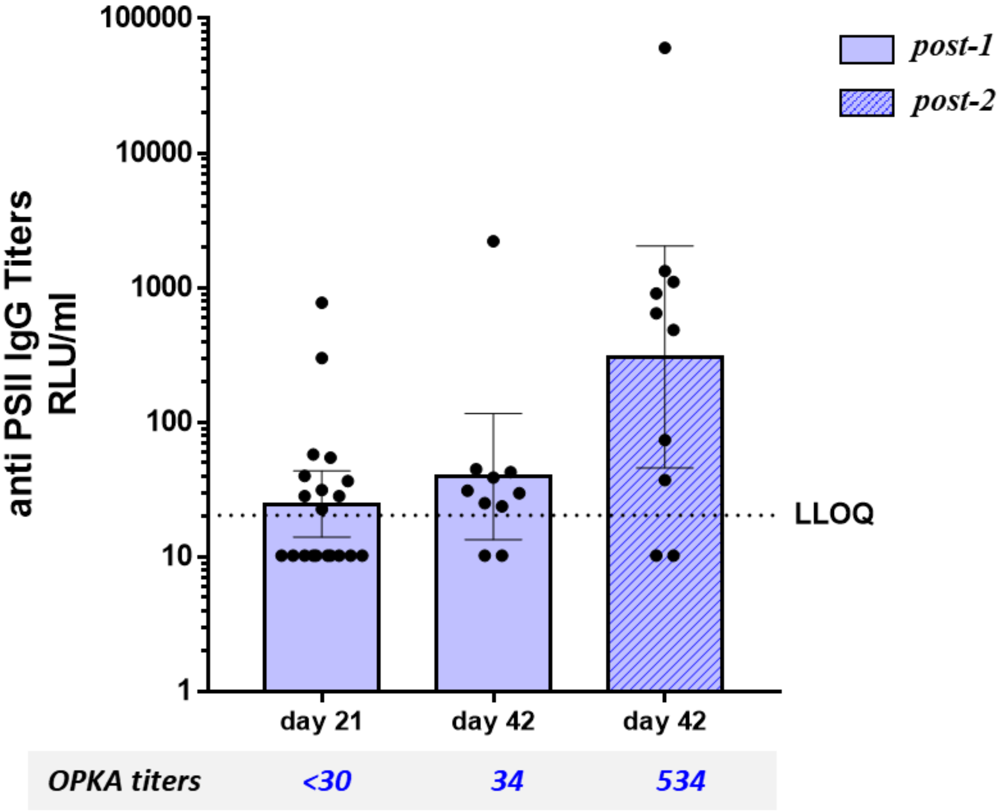

Supplementary Figure 2. Antibody responses in mice receiving GBS PSII-CRM after 1 and 2 doses. PSII IgG titers in serum samples collected from mice (10 per group) receiving 1 (full bars) or 2 doses (patterned bar) of PSII-CRM. The geometric mean titer (RLU/mL) is indicated by the bars, individual mice are indicated by the dots, and the 95% Confidence Interval is indicated by the wiskers. For GMT, non responder sera were assigned titers half of the LLOQ. The corresponding OPKA titers from pools of serum samples are reported below the barchart.

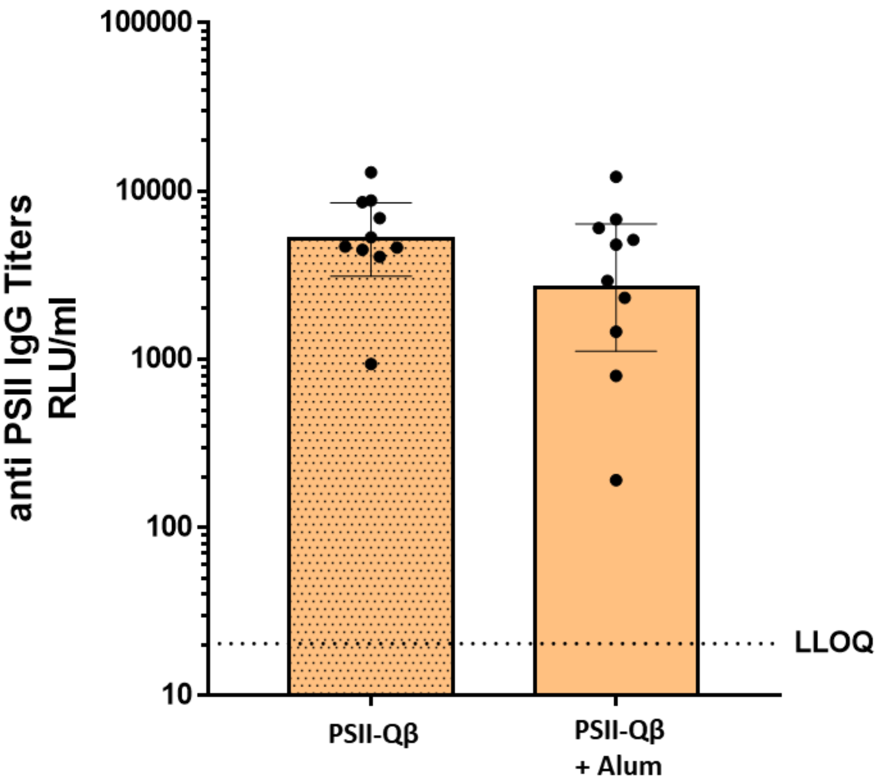

OPKA titers	*855*	*980*

Supplementary Figure 3. Antibody responses in mice receiving 2 doses of GBS PSII-Qβ with or without Aluminum hydroxide. PSII IgG titers in serum samples collected from mice (10 per group) receiving 2 doses of PSII-CRM. The geometric mean titer (RLU/mL) is indicated by the bars, individual mice are indicated by the dots, and the 95% Confidence Interval is indicated by the wiskers.. The corresponding OPKA titers from pools of serum samples are reported below the barchart.

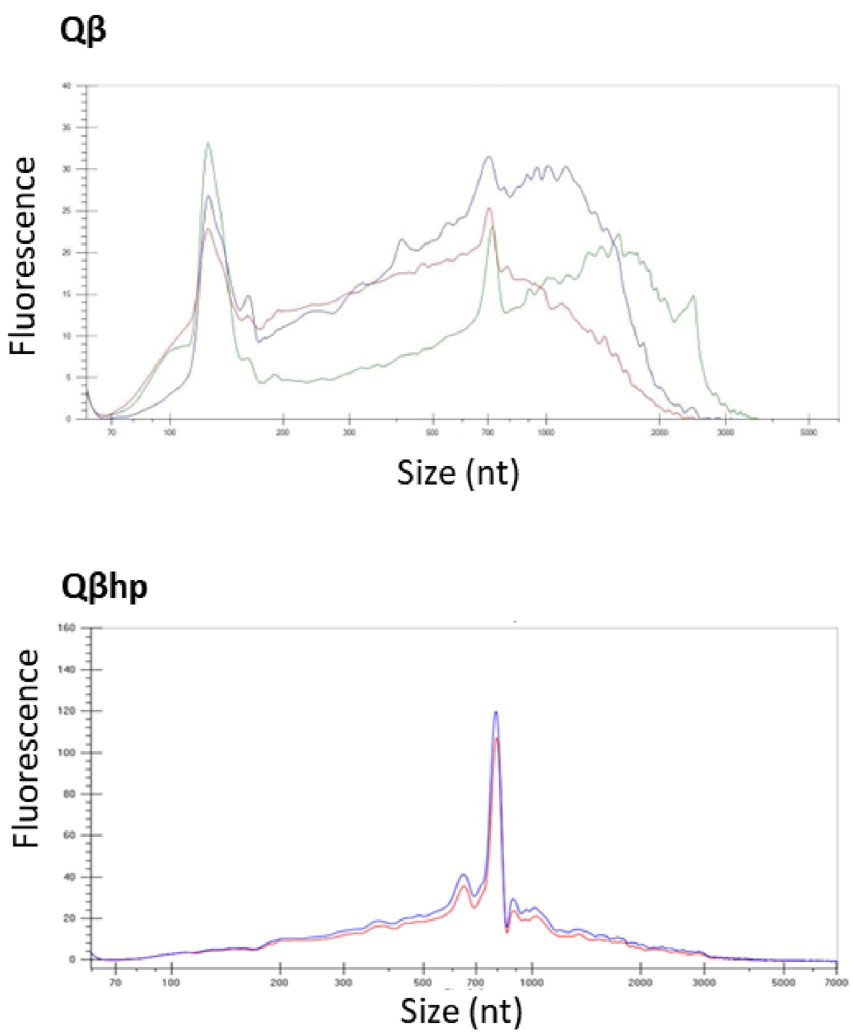

Supplementary Figure 4. Capillary electrophoresis analysis of RNA extracted from Qβ and Qβhp.

LabChip GX II analysis on RNA from 3 lots of Qβ (top) and 2 lots of Qβhp (bottom)
